# Comparing Smartphone Apps for Traditional Chinese Medicine and Modern Medicine in China: Systematic Search and Content Analysis

**DOI:** 10.2196/27406

**Published:** 2021-03-24

**Authors:** Xiao Hang Liu, Fan Jin, Jeffrey Hsu, Di Nan Li, Wei Chen

**Affiliations:** 1 Department of Cardiology Peking Union Medical College Hospital Chinese Academy of Medical Sciences & Peking Union Medical College Beijing China; 2 Department of Infectious Diseases Peking Union Medical College Hospital Chinese Academy of Medical Sciences & Peking Union Medical College Beijing China; 3 Yawlih Technology Beijing China

**Keywords:** mHealth, traditional Chinese medicine, modern medicine, mobile apps, app, comparison, content analysis, China, health care, development

## Abstract

**Background:**

Traditional Chinese medicine (TCM) is an integral part of mainstream medicine in China, with theories and practices that are completely different from modern medicine. TCM should not be ignored or confused with modern medicine in the analysis of the Chinese health care system, including the analysis of mobile health (mHealth) apps. To date, differences between TCM apps and modern medicine apps have not be systematically investigated.

**Objective:**

The aim of this study was to systematically compare the quality of apps for TCM and modern medicine in China.

**Methods:**

In December 2020, we searched iOS (iTunes) and Android (Tencent, Oppo, and Huawei app stores) platforms for all mHealth apps and then categorized them as TCM or modern medicine apps if they were included in the final analysis. The included apps were downloaded on smartphones and assessed by 2 reviewers on the following 4 aspects: (1) data in the app stores, including user ratings, download counts, cost, target users, and year of last update; (2) functionality; (3) quality of the app content as determined by the Mobile App Rating Scale (MARS); and (4) analysis of the app privacy and security.

**Results:**

In total, 658 apps were analyzed, including 261 TCM medicine apps and 397 modern medicine apps. The average download count of modern medicine apps (approximately 5 million) was more than 10 times that of TCM apps (approximately 400,000). Regarding functionalities, 64.7% (257/397) of modern medicine apps provided telemedicine (74/261, 28.4% in TCM apps), 62.7% (249/397) provided registration (70/261, 26.8% in TCM apps), and 45.6% (181/397) provided communication (38/261, 14.6% in TCM apps). A larger proportion of TCM apps provided prescription and medication management (144/261, 55.2% in TCM apps versus 168/397, 42.3% in modern medicine apps). The majority of modern medicine apps (329/397, 82.9%) combined ≥3 functionalities compared with one-third of TCM apps (93/261, 34.6%). We then selected 81 top apps for quality and safety assessment (41 TCM apps and 40 modern medicine apps). Of these, the mean overall MARS score of TCM apps (2.7, SD 0.5) was significantly lower than modern medicine apps (3.6, SD 0.4). Almost all modern medicine apps (38/40, 95%) addressed privacy and security by providing a privacy policy and describing how to protect personal data, but less than half of the TCM apps (18/41, 44%) described this information (*P*<.001).

**Conclusions:**

The different functionalities reflect the distinct innate characteristics of these two medical systems. Although great progress has been made and the Chinese mHealth market size is large, there still exist many opportunities for future development, especially for TCM.

## Introduction

Traditional Chinese medicine (TCM) refers to a holistic medical system for the pathophysiology, diagnosis, treatment, and prevention of diseases [1]. As one of the oldest traditional medicine systems in the world, TCM was formed more than 2000 years ago and developed with the accumulation of knowledge and practice in the following centuries [2-5]. Outside China, TCM is treated as an important part of complementary and alternative medicine and has gained increasing attention [6]. In 2018, the World Health Organization (WHO) first included a chapter on TCM in the 11th revision of the International Classification of Diseases [7]. In China, with strong support from the government for the popularization of TCM and people’s belief in traditional Chinese culture, there is no doubt that TCM is an integral part of mainstream medicine. According to the National Health Commission of the People’s Republic of China, there were nearly 35,000 hospitals in 2019 in China, and 15.2% (5232) of them were hospitals of TCM. Meanwhile, there were 3267 outpatient departments and over 57,000 clinics of TCM. The TCM sector provided more than 1.1 billion medical services, accounting for 16.4% of health care in China [8]. During the COVID-19 pandemic, TCM also played an active role in the prevention of SARS-CoV-2, helped improve clinical symptoms of patients, and reduced the mortality rate [9].

The rapid development of information and communication technologies helps overcome the barrier of distance in the delivery of health care services, which enables the generation and proliferation of mobile health (mHealth) [10]. mHealth apps have epitomized typical mHealth service and have the potential to promote patient engagement, cut costs, and improve health outcomes [11]. In recent years, the establishment of telecommunication networks, continuous growth of smartphone usage rates, and increasing demand for high-quality and convenient health care services has led to significant development of the mHealth industry in China, including numerous and miscellaneous apps released. In 2020, the market size of mHealth reached ¥52.1 billion (approximately US $8.04 billion), and the number of mHealth users was 6.35 billion [12]. So far, several investigations providing an overview of the characteristics of mHealth apps in China have been published, but they did not distinguish whether the apps were tailored for TCM or modern medicine [13,14]. Given the unique medical system in China and natural differences in many aspects of TCM and modern medicine, including mechanisms of actions, mode of treatment, training of practitioners, quality of medicines, involvement of the healer and the patient, safety, and adverse effects [2], the performance and contribution of TCM should not be ignored or confused with modern medicine (also known as Western medicine in China) in the analysis of mHealth apps.

Thus, the aim of this study was to systematically compare the quality of apps for TCM and modern medicine in China. Specific objectives were to assess the basic characteristics, functionalities, app content (using the Mobile App Rating Scale [MARS]), and fairness of privacy policies. To our knowledge, no similar study has been done.

## Methods

### Search Criteria and App Selection

In December 2020, we conducted a thorough review of mHealth apps across the Apple iTunes app store (iOS). For Android, we sampled apps from the 3 largest Android app stores in China: Tencent Myapp, Oppo, and Huawei [15]. Unlike most other countries, the Google Play Store was blocked in China and the general public could not access Google Play and download apps. Therefore, we searched the most commonly used Chinese app stores for Android devices to reflect the real-world conditions in China. The following search terms were identified: “mobile health” OR “medicine” OR “traditional Chinese medicine” OR “traditional medicine” OR “modern medicine” OR “Western medicine.” There were no restrictions concerning subcategories like “medical.”

Screening was conducted based on app titles, marketing descriptions, and screenshots of the potential apps for relevance and inclusion. The following apps were excluded: (1) apps not relevant to our study purpose or designed for entertainment, product advertisement, loans, etc; (2) apps not in Chinese; (3) question banks or online guidance for examinations like the medical licensing examination, professional postexamination, and test for the national residency standard training program; (4) apps focused on hospital administration and management; and (5) apps pertaining to general health, for example, menstrual cycle management, sleep monitoring, and water intake reminder apps. The remaining apps were downloaded for eligibility. If an app could not run properly, it was also excluded. After selection, the included apps were classified as TCM apps or modern medicine apps based on the app’s content. All apps were downloaded onto an iPhone 12 (version 14.2.1) and a Huawei Nova 2s (version 9.0). Two reviewers (XHL and FJ) performed the assessment of the apps using a standard data extraction form.

### Assessment of Apps

First, the general characteristics of the apps were recorded in the database, including platform, average user-scored star rating, download counts, year of the last update, target user, and app cost. Then, we investigated the services each app provided through use of the app. The specific functionalities used in this study were telemedicine, registration, prescription and medication management, communication, records, citizen-based reporting, on-demand information services to clients, client financial transactions, decision support, health worker activity planning and scheduling, health care provider training, and laboratory and diagnostics imaging management. The definition of these functionalities originated from the classification of digital health interventions proposed by the WHO and were tailored to this study [16,17].

For further analysis, we selected the top 25 Android apps and top 25 iOS apps from TCM and modern medicine apps, respectively. For Android, we chose by the number of downloads in descending order. For iOS, as information on the number of downloads was not provided, the order of selection was dependent on search retrieval order on the platform.

The MARS was then used to rate the selected apps’ quality, including objective and subjective app quality evaluation [18]. The objective app quality section contained 19 evaluation criteria clustered within 4 domains: engagement, functionality, aesthetics, and information. The domain of subjective quality included 4 criteria to evaluate the overall satisfaction of users. Each evaluation criterion was scored on a 5-point Likert scale (1=unqualified, 2=poor, 3=acceptable, 4=good, 5=excellent). Two independent reviewers viewed the training video and tested each app for at least 10 minutes. After scoring all the evaluation criteria, the total mean MARS score describing the overall quality of the app was obtained by calculating the average value of the 5 domains.

Additionally, based on the guidance of privacy in mobile apps recommended by the Information Commissioner’s Office [19] and the mobile app privacy and security best practices published by the Online Trust Alliance [20], the assessment of privacy and security consisted of 7 questions. By answering yes or no, the accessibility of the privacy policy and ability to protect personal data were assessed.

Any discrepancies in the assessment of apps were resolved by discussion with other researchers (DNL and JH) of the study team until consensus was reached.

### Analysis of Apps Operated by Chinese Top Hospitals

The ranking list of Chinese hospitals is published every year to provide direction for discipline construction and guidance for patients [21,22]. Hospitals are rated on medical quality, resource allocation, academic research, and other criteria. In the apps fulfilling the inclusion criteria of our study, we first identified the number of apps operated by the top 100 TCM hospitals and top 100 modern medicine hospitals, respectively, according to the latest ranking list. Because appointment scheduling is the main function of apps directly operated by hospitals, we then investigated whether the patients needed to verify their identity before making an appointment and whether the apps provided appointment guidance, doctor selection, department selection, and online payment services during appointment scheduling.

### Statistical Analysis

Categorical variables were compared using an uncorrected chi-square test or Fisher exact test. Continuous variables were analyzed using independent *t* tests. In the MARS evaluation, the Cohen test was performed to guarantee the reliability of the data analyzed by the 2 independent researchers. All statistical analyses were performed using IBM SPSS 22.0 (IBM Corp). A *P* value of <.05 was considered statistically significant.

## Results

### Summary of Search Results

**Figure 1:**
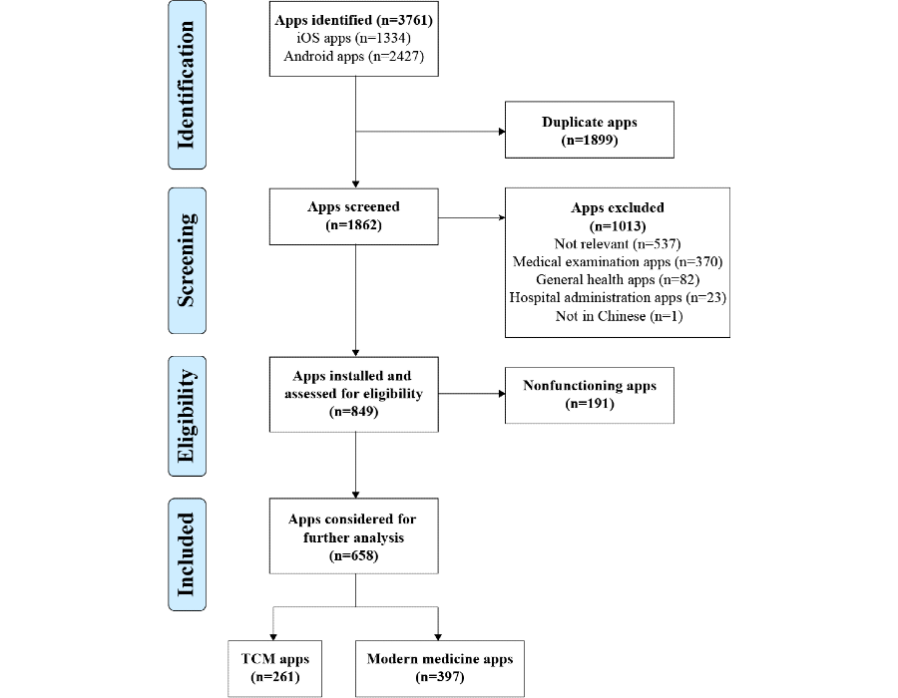
Screening process flowchart. TCM: traditional Chinese medicine.

iOS and Android app store searches identified 3761 potential apps, of which 1899 were removed as duplicates. Of the remaining 1862 apps, 658 met the indicated criteria. Among these included apps, 261 apps were classified as TCM apps and the other 397 focused specifically on modern medicine. The flow diagram (Figure 1) provides an overview of the selection process and reasons for exclusion. Major reasons for exclusion were that the app was not relevant to medicine (n=537) or it was a medical examination app (n=370).

### General Characteristics of Apps

Table 1 summarizes the general characteristics of the included TCM apps and modern medicine apps. There was no difference in overall user-scored rating between these two categories of apps (4.0, SD 1.1 vs 3.8, SD 1.2; *P*=.11). Although the overall user ratings were generally equal, modern medicine apps had a huge advantage in the number of downloads. In Tencent Myapp, the average number of downloads of modern medicine apps (approximately 5 million) was more than 10 times that of TCM apps (approximately 400,000). In addition, modern medicine apps were updated more frequently. A larger percentage of modern medicine apps were updated during the last year (352/397, 88.7% of modern medicine apps compared with 151/261, 57.9% of TCM apps), while 10.7% (28/261) of TCM apps had not been updated in the last 3 years. Most apps could be downloaded without cost, including 98.7% (392/397) of modern medicine and 91.2% (238/261) of TCM apps. There was a large difference in the constitution of target users. A total of 74.7% (195/261) of the TCM apps could be used by people who were not health care professionals (HCPs), but 41.6% (165/397) of the modern medicine apps were designed only for HCPs.

**Table 1:** General characteristics of TCM apps and modern medicine apps.

Characteristics			TCM^a^ (n=261)		Modern medicine (n=397)		*P* value
**Platform, n (%)**							
	iOS	109 (41.8)		94 (23.7)		<.001	
	Android	115 (44.1)		236 (59.4)		<.001	
	iOS and Android	37 (14.2)		67 (16.9)		.35	
**Ratings, mean (SD)**							
	iOS	4.0 (1.1)		4.5 (0.7)		<.001	
	Android	3.6 (1.3)		3.2 (1.3)		.03	
	iOS and Android^b^	4.4 (0.7)		4.5 (0.4)		.26	
	Overall	4.0 (1.1)		3.8 (1.2)		.11	
**Number of downloads^c^ (thousand), n (%)**							
	>10,000	0 (0)		24 (7.9)		<.001	
	1000-9999	10 (6.6)		54 (17.8)		<.001	
	100-999	39 (25.7)		110 (36.3)		<.001	
	10-99	54 (35.5)		88 (29.0)		.65	
	<10	49 (32.2)		27 (8.9)		<.001	
**Year of last update, n (%)**							
	2020	151 (57.9)		352 (88.7)		<.001	
	2019	42 (16.1)		27 (6.8)		<.001	
	2018	18 (6.9)		9 (2.3)		.003	
	Before 2018	28 (10.7)		5 (1.3)		<.001	
	No updates	22 (8.4)		4 (1.0)		<.001	
**Cost, n (%)**							
	No	238 (91.2)		392 (98.7)		<.001	
	Yes	23 (8.8)		5 (1.3)		<.001	

^a^TCM: traditional Chinese medicine.

^b^Ratings were calculated as the average on iOS and Android platforms.

^c^We only counted the number of downloads on Android because this was not applicable on iOS. TCM: n=152; modern medicine: n=303.

### Functionalities of Apps

**Figure 2:**
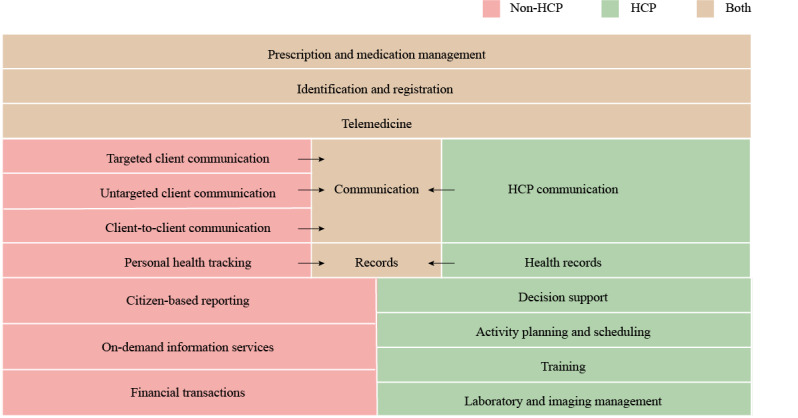
Classification criteria of functionalities. The criteria originated from the classification of digital health interventions proposed by the WHO and were tailored to this study. As opposed to the WHO criteria, prescription and medication management, identification and registration, and telemedicine were not under a single category of HCP or non-HCP because they could be mutual processes. Targeted client communication, untargeted client communication, client-to-client communication, and HCP communication were summarized as "communication." “Records” included personal health tracking and health records. Referral coordination was removed from our study because no app provided this service. HCP: health care professional; WHO: World Health Organization.

The detailed classification criteria of functionalities are shown in Figure 2. TCM and modern medicine apps displayed divergent functionalities (as shown in Table 2). As a whole, the most common service factors were telemedicine, identification and registration, and prescription and medication management. The least common service factors were laboratory and imaging management and activity planning and scheduling. By comparison, more modern medicine apps provided services such as registration, telemedicine, and communication. A larger proportion of TCM apps provided prescription and medication management. Specifically, not only were herbs and Chinese patent drugs available in the online pharmacy of TCM apps but the service of daily home delivery of decocted drugs was also provided by these apps because some herbs need to be boiled. Similar proportions of TCM and modern medicine apps provided on-demand information services for non-HCPs. Regarding the content of the information services, we noticed that many TCM apps provided relatively professional knowledge and self-screening. Professional knowledge of TCM was mainly conveyed by presenting and interpreting the classic TCM books, like *Inner Canon of the Yellow Emperor* (earliest medical classic in China) and *Compendium of Materia Medica* (an outline treatise of medical herbs). They provided e-books or audio files. Self-screening was usually done by answering questionnaires that showed the patients whether they had a yin deficiency or yang deficiency of certain organs. Using artificial intelligence, one app analyzed the health condition of a user’s body after capturing and uploading their facial expression (Multimedia Appendix 1).

**Table 2:** Frequency of app functionalities.

Functionality	Total, n (%) (n=658)	TCM^a^, n (%) (n=261)	Modern medicine, n (%) (n=397)	*P* value
Prescription and medication management	312 (47.4)	144 (55.2)	168 (42.3)	.001
Identification and registration	319 (48.4)	70 (26.8)	249 (62.7)	<.001
Telemedicine	331 (50.3)	74 (28.4)	257 (64.7)	<.001
Communication	219 (33.3)	38 (14.6)	181 (45.6)	<.001
Records	149 (22.6)	24 (9.2)	125 (31.5)	<.001
Citizen-based reporting	160 (24.3)	56 (21.5)	104 (26.2)	.16
On-demand information services	286 (43.4)	106 (40.6)	180 (45.3)	.23
Financial transactions	210 (31.9)	62 (23.8)	148 (37.3)	<.001
Decision support	164 (24.9)	43 (16.5)	121 (30.5)	<.001
Activity planning and scheduling	54 (8.2)	12 (4.5)	42 (10.5)	.006
Training	130 (19.8)	52 (19.9)	78 (19.6)	.93
Laboratory and imaging management	46 (6.9)	10 (3.8)	36 (9.0)	.01

^a^TCM: traditional Chinese medicine.

**Figure 3:**
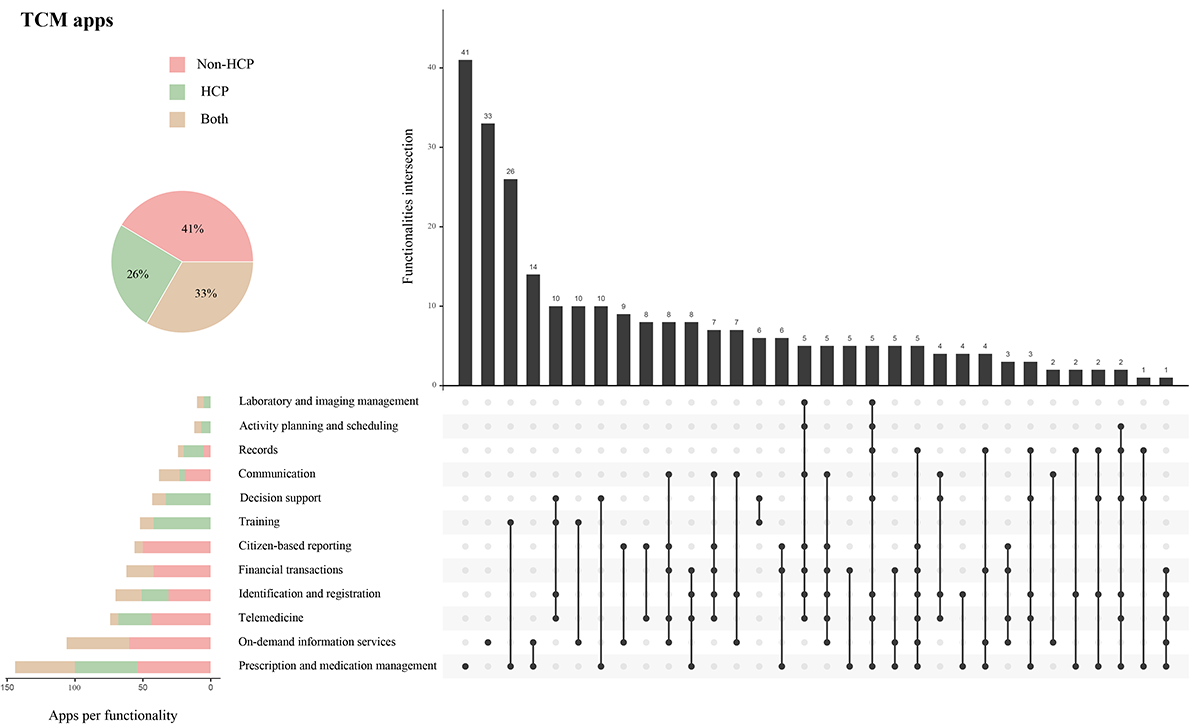
TCM apps’ functionalities. The pie chart shows the percentage of TCM apps’ target users. The stacked bar chart shows the distribution of TCM apps according to key functionalities and user type. The upset plot shows the intersection of multiple functionalities. TCM: traditional Chinese medicine.

**Figure 4:**
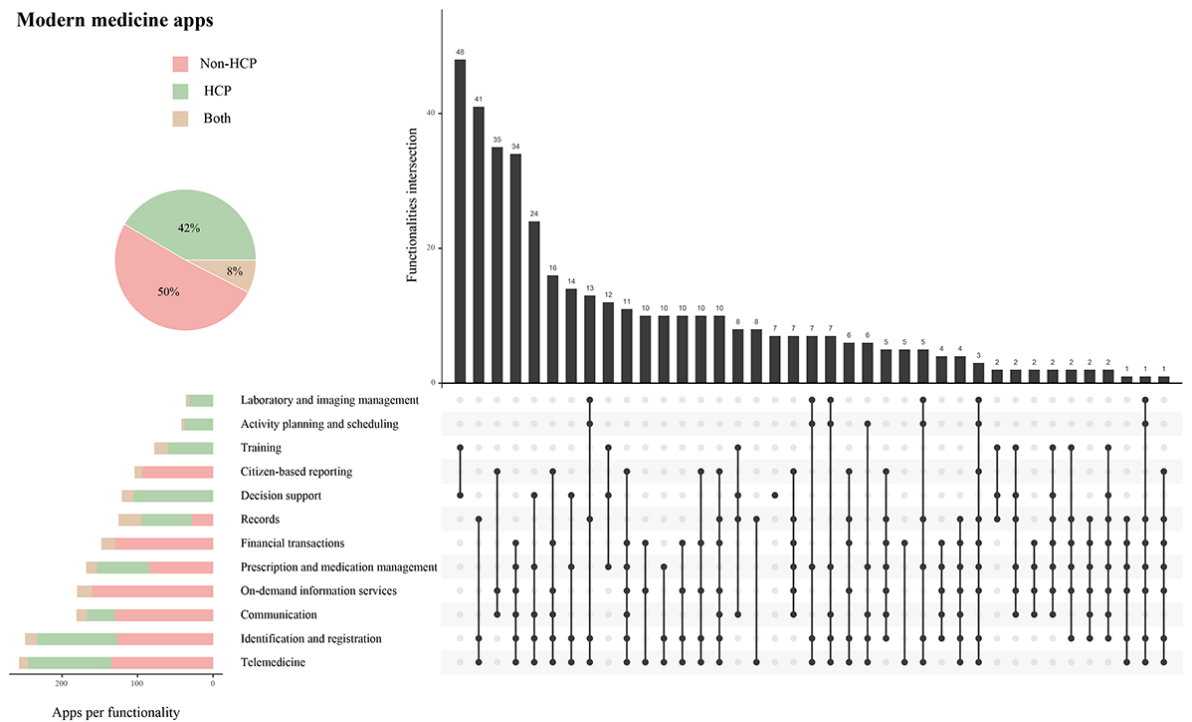
Modern medicine apps’ functionalities. Pie chart: portion of modern medicine apps’ target users. Stacked bar chart: distribution of modern medicine apps according to key functionalities and user type. Upset plot: intersection of multiple functionalities.

We further analyzed the combinations of app functionalities. Figures 3 and 4 show all the combinations of TCM apps and modern medicine apps. Compared with TCM apps, modern medicine apps provided more comprehensive functionalities and various combinations. The majority of modern medicine apps (329/397, 82.9%) combined ≥3 functionalities compared with about one-third of TCM apps (93/261, 35.6%). Modern medicine apps also produced more combination patterns than TCM apps (39 vs 32). The most common combinations of functionalities in TCM apps were (1) prescription and medication management plus training and (2) prescription and medication management plus on-demand information services, while the most common combinations of functionalities in modern medicine apps were (1) decision support plus training and (2) telemedicine plus identification and registration plus records.

### MARS Evaluation of Top Apps

Among the top 50 apps of the two categories, 9 TCM apps and 10 modern medicine apps were available on both iOS and Android platforms. Therefore, 41 TCM apps and 40 modern medicine apps were included in the MARS evaluation.

The mean overall MARS score for the TCM apps was significantly lower than that for the modern medicine apps (2.7, SD 0.5 vs 3.6, SD 0.4; *P*<.001) (Table 3). The percentage of apps that scored higher than the minimum acceptability score of 3.0 was 34% (14/41) of TCM apps and 95% (38/40) of modern medicine apps. A total of 5 TCM apps scored ≤2 points and none scored ≥4 points. By contrast, among the modern medicine apps, no apps scored ≤2 points and 4 scored ≥4 points.

**Table 3:** Comparison of TCM and modern medicine apps’ results for the Mobile App Rating Scale evaluation.

Category	TCM^a^, mean (SD) (n=41)	Modern medicine, mean (SD) (n=40)	*P* value
Engagement	2.3 (0.5)	3.2 (0.5)	<.001
Functionality	3.5 (0.6)	4.0 (0.3)	<.001
Aesthetics	2.6 (0.8)	3.8 (0.5)	<.001
Information	2.9 (0.5)	3.7 (0.4)	<.001
Subjective quality	2.2 (0.7)	3.4 (0.8)	<.001
Overall	2.7 (0.5)	3.6 (0.4)	<.001

^a^TCM: traditional Chinese medicine.

**Figure 5:**
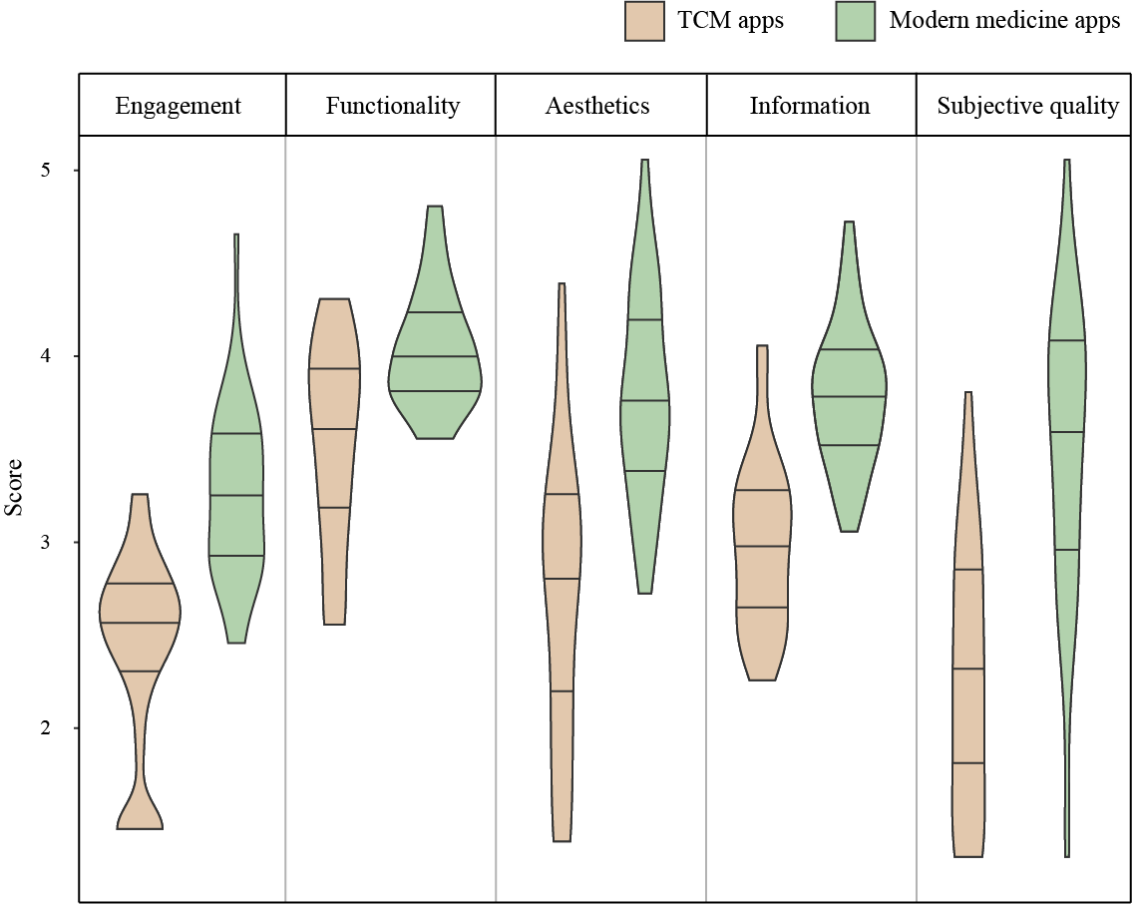
Violin plot of the Mobile Application Rating Scale section item scores. The 3 black lines in each plot present the first quartile, the median, and the third quartile (from bottom to top). TCM: traditional Chinese medicine.

In all 5 domains (engagement, functionality, aesthetics, information, and subjective quality), modern medicine apps outperformed TCM apps, and the differences were statistically significant (*P*<.001). Functionality had the highest mean score in both TCM apps (3.5, SD 0.6) and modern medicine apps (4.0, SD 0.3), while engagement was the poorest in the objective quality for both kinds of apps (3.2, SD 0.5 in modern medicine; 2.3, SD 0.5 in TCM). The Cohen coefficient was 0.92, indicating excellent interrater reliability. Figure 5 presents the specific score distribution of the two categories of apps in each domain.

### Privacy and Security of Top Apps

The privacy policy was available for 68% (28/41) of TCM apps and 95% (38/40) of modern medicine apps (*P=.*02). In comparison with TCM apps, more modern medicine apps (17/40, 43% vs 6/41, 15%; *P*=.008) had a short-form privacy and security notice that highlighted key data practices. More modern medicine apps collected personally identifiable information and shared the data with third parties. Additionally, modern medicine apps performed better in data safety protection. A total of 95% (38/40) of the modern medicine apps described how the personal data were protected, but only 44% (18/41) of TCM apps described this information (*P*<.001) (Table 4).

**Table 4:** Assessment of privacy and security in regard to data gathering, sharing, and security as described in the privacy policy.

Privacy and security question		Total, n (%) (n=81)	TCM^a^, n (%) (n=41)	Modern medicine, n (%) (n=40)	*P* value
**Is the privacy policy available without the need to download the app?**					.02
	No	15 (18.5)	13 (31.7)	2 (5.0)	
	Yes	66 (81.5)	28 (68.3)	38 (95.0)	
**Is the privacy policy available within the app?**					.004
	No	24 (29.6)	18 (43.9)	6 (15.0)	
	Yes	57 (70.4)	23 (56.1)	34 (85.0)	
**Is there a short-form notice highlighting key data practices?**					.008
	No	48 (59.3)	27 (65.9)	21 (52.5)	
	Yes	23 (28.4)	6 (14.6)	17 (42.5)	
	Not applicable	10 (12.3)	8 (19.5)	2 (5.0)	
**Is the privacy policy available in any other language?**					.23
	No	78 (96.3)	41 (100)	37 (92.5)	
	Yes	3 (3.7)	0 (0)	3 (7.5)	
**Does the app collect personally identifiable information?**					.004
	No	14 (17.3)	12 (29.3)	2 (5.0)	
	Yes	65 (80.2)	27 (65.9)	38 (95.0)	
	Not specified	2 (2.5)	2 (4.9)	0 (0)	
**Does the app share users' data with a third party?**					<.001
	No	16 (19.8)	11 (26.8)	5 (12.5)	
	Yes	44 (54.3)	12 (29.3)	32 (80.0)	
	Not specified	21 (25.9)	18 (43.9)	3 (7.5)	
**Does the app say how the users' data security is ensured (eg, encryption, authentication, fire wall)?**					<.001
	No	25 (30.9)	23 (56.1)	2 (5.0)	
	Yes	56 (69.1)	18 (43.9)	38 (95.0)	

^a^TCM: traditional Chinese medicine.

### Comparison of Hospital Apps

Among the top hospitals in China, 41 hospitals of modern medicine had their own apps, but the number sharply decreased to 11 for hospitals of TCM. As expected, all these apps provided the service of registration. Although significantly fewer hospitals of TCM developed apps for patients, regarding the procedure of appointment scheduling, all hospital apps, no matter if they were TCM or modern medicine apps, provided the services of department selection and doctor selection so that the patients could visit specific doctors according to the patients’ preferences and the doctors’ areas of expertise. Identity verification was also required in all apps via identity card or phone number. This could help stop scalpers making profits from scheduling fake appointments. Due to the rapid development of e-commerce in China, all TCM apps and 33 of 41 (81%) modern medicine apps allowed online payment via Alipay, Wechat, and debit card. A total of 78% (32/41) of modern medicine apps and 64% (7/11) of TCM apps provided appointment guidance, but the difference was not statistically significant (*P*=.56).

## Discussion

### Principal Findings

This study systematically compared the apps designed for TCM and modern medicine available in China. Considering that TCM is a traditional medicine system and mHealth is a newly developed information technology and taking into account the important role of TCM in Chinese culture and the Chinese medical system, TCM should not be overlooked or simply confused with modern medicine in the analysis of mHealth apps. Overall, our findings suggest that there are currently a considerable number of both TCM (n=261) and modern medicine (n=397) apps on the market, but TCM apps and modern medicine apps had distinct functionalities and combinations of functionalities. The TCM apps scored lower in all aspects (engagement, functionality, aesthetics, information quality, and subjective quality) of the multidimensional measure of app quality using the MARS app rating tool. Great progress has been made regarding the privacy and security of mHealth apps in China. We discuss our findings further below.

The Android app stores with the largest number of monthly active users in China are operated by Tencent, Oppo, and Huawei. Google Play is difficult to access and has been almost absent since 2010 [13,23]. Thus, we sampled apps from Tencent Myapp, Oppo App Store, Huawei App Store, and Apple iTunes App Store and applied keywords commonly used for TCM and modern medicine to ensure the representativeness of the apps we included. Our study displayed a current landscape of 261 TCM apps and 397 modern medicine apps. More than half of the modern medicine apps had been updated in the last year to improve performance, fix bugs, and update information to adapt to rapidly growing medical knowledge. TCM apps and modern medicine apps had similar overall ratings, but download counts of modern medicine apps were much higher. Nearly 10% of modern medicine apps had over 10 million downloads, and the top app, named Ping An Good Doctor, had more than 200 million downloads. In contrast, more than half of TCM apps did not reach the download volume of 100,000. This result indicates that although a certain number of TCM apps have been developed, the subsequent promotion, usage, and software maintenance remain problems. The majority of people are used to the traditional offline modality of TCM rather than the use of mHealth apps.

As displayed in the functionalities and combinations of functionalities, both TCM and modern medicine apps attached great importance to on-demand information services to clients. The differences in functionalities reflected the differences in medical theories and practices between TCM and modern medicine. The mechanism of modern medicine is based on anatomy, pathophysiology, molecular biology, and other basic medical and clinical medical knowledge, while the formation of TCM is on the basis of ancient Chinese philosophy. The diagnosis of a certain disease in Western medicine needs modern equipment and laboratory testing, while diagnosing a *zheng* in TCM relies more on the doctor’s experience and observation without drawing blood or doing radiological examinations [24]. A *zheng* (syndrome) is an outcome after analyzing all symptoms and signs. One disease in modern medicine may have several *zheng* and a *zheng* could be caused by different diseases [25]. Modern medicine primarily treats patients through medicine or surgery with additional information about precautions and side effects, while TCM treatment approaches include herbs, minerals, and guidelines on lifestyle [26]. Therefore, preliminary understanding of TCM is easier for the public, and the gap between HCPs and non-HCPs is smaller. As a result, modern medicine apps pay more attention to building connections between doctors and patients, while TCM apps tend to educate the patients more. In our study, 41.6% (165/397) of modern medicine apps were designed for HCPs, while 74.7% (195/261) of TCM apps’ target users included non-HCPs.

Similarly, more modern medicine apps provided telemedicine and appointment-making services. In China, patients can purchase Chinese patent drugs without strict restrictions, which is much easier than buying prescription drugs. This explains why medication management appeared more commonly in TCM apps. Because laboratory tests and imaging examinations are important parts of modern medicine, it was no surprise that laboratory and diagnostics imaging management services were more common in modern medicine apps.

We extracted top TCM and modern medicine apps for further analysis of quality and security. MARS is a systematic and validated questionnaire that is not too technical or specific to a particular health domain [18,27]. Our study showed a mean score of 2.7 and 3.6 for the overall quality of TCM apps and modern medicine apps, respectively. A rating of ≥3 points indicates overall acceptable quality [28]. All top modern medicine apps scored a value of ≥3 points, and 4 of 41 (10%) apps exceeded 4 points. However, half of TCM apps did not reach the acceptable level. The score of Western medicine apps was similar or even higher compared with the results of other studies. Davalbhakta et al [29] showed a rating of 3.7 in apps for the management of COVID-19. Kim et al [30] found the mean MARS score for the overall quality of apps to be 3.23 for potential drug-drug interaction checks. TCM apps scored much lower in both objective and subjective quality assessments. Differences between TCM apps and modern medicine apps were statistically significant for all dimensions of the MARS. The engagement domain scored poorest in both categories, which is consistent with previous studies of apps targeting genitourinary tumors and COVID-19 [29,31]. This could be explained by the primary purpose of mHealth apps. Because the main target users of mHealth apps are either doctors or patients, it is difficult to make them feel interested and relaxed when facing diseases. Apps focusing on behavior change might score higher on the engagement dimension because the engagement of users is a key factor of successful behavior change [32].

Privacy policies and data security are particularly important for mHealth apps because they usually collect personally identifiable information and data related to the users’ health conditions. It is disappointing that privacy policies were absent within and outside of the app (on the website or app store) in 9 of the top 41 (22%) TCM apps and that 23 of the 41 (56.1%) apps did not mention how they ensured the users’ data security at all. However, we believe this is a positive fact for two reasons. First, according to Hsu et al [13], in December 2015, nearly all Chinese top mHealth apps, let alone the remaining apps, lacked information security. The Chinese government did not have a direct policy or documents on mHealth security then [13]. In June 2016, the general office of the People’s Republic of China State Council issued guidance on promoting and standardizing the development of health care applications. One of the major principles was keeping a balance between app development and its safety and protecting individual privacy and information security effectively [33]. It is encouraging that great progress has been made in recent years, starting from scratch. Second, we included various types of apps in our study. The main function of 6 of the 9 apps without privacy policies was health care information provision to clients, and they did not collect personally identifiable information. Likewise, Sunyaev et al [34] found a surprising result in that only 31% of medical or health and fitness apps had privacy policies. Privacy policies might be less frequently provided in apps handling less sensitive information about patients. In terms of modern medicine apps, all of them incorporated or linked to a privacy policy, which is even better than the findings of Huckvale et al [35], who studied top-ranked apps for depression and smoking cessation on Android and iOS platforms. They assessed privacy policies in a more detailed way according to a schema of privacy policy quality criteria [36]. In this study, we managed to provide a comprehensive comparison between TCM apps and modern medicine apps instead of deeply investigating their privacy policies.

### Future Perspectives

There exist many opportunities for further development of both TCM and modern medicine apps in China in the future. On the one hand, for TCM, mHealth is a perfect tool that has not been fully used. The basic diagnostic procedure of TCM is composed of 4 techniques: looking, listening and smelling, questioning, and feeling the pulse. The first 3 methods can be easily realized using photos and videos via remote communication technologies on mHealth apps. Moreover, Tang et al [37] reported an electronic TCM pulse diagnostic system developed with an artificial neural network. Considering that herbs and Chinese patent drugs are not strictly restricted and there is a well-developed express industry in China, diagnosing and treating without seeing patients face to face is more realizable for TCM. TCM hospitals should have made more efforts to develop online services, but in our study, only 11 of the top 100 TCM hospitals had their own apps in service. In addition, in order to make TCM information more evidence based and accepted both domestically and internationally, the Chinese government and TCM experts should keep investing in programs devoted to the modernization and standardization of TCM. On the other hand, for modern medicine, the online-to-offline approach (an integration of offline businesses into online commerce) has been relatively mature [38]. In the future, internet hospitals should be further developed to break the reliance on traditional health care providers [39].

### Limitations

There are limitations to this study. First, we did not access Google Play for the Android apps, which could cause selection bias. Google Play is widely used worldwide. However, the general public in mainland China could not download or purchase apps on Google Play at all. Therefore, we selected the 3 largest Android app stores in China to reflect the real-world conditions [15]. Second, sometimes it was difficult to put an app into the category of TCM or modern medicine because the apps (less than 10 in our study) provided both kinds of services simultaneously. We fully assessed all the functions and discussed to determine the main focus of these apps. If disagreement existed, a third experienced investigator was invited. Third, we excluded general health apps to achieve a more homogeneous analysis because we could not define whether general health apps belonged to TCM or modern medicine. Finally, under the category of both TCM and modern medicine, we presented broad coverage and apps for various initiatives were included. It is worth mentioning that the main purpose of this study was to provide a global analysis of the distribution of and differences between TCM apps and modern medicine apps rather than apps for a single disease or service.

### Conclusions

We identified the number and functions and evaluated the quality and privacy of TCM apps and modern medicine apps currently available on the Chinese market. mHealth in China is already a large market for both TCM and modern medicine, but there is still great potential for development. Different functionalities reflected the distinct innate characteristics of these two medical systems. Apps for modern medicine outperformed TCM apps in all aspects of quality assessment using MARS. TCM apps need quality improvement for further penetration into the market. It is gratifying that the developers, data controllers, and government paid attention to the privacy and security of mHealth apps. This work can inform the future development of mHealth apps for developers and provide an important reference for researchers and customers to search, review, and compare the TCM and modern medicine apps in China.

## Appendix

Multimedia Appendix 1 Screenshot of a TCM app for self-screening.

## Abbreviations

HCP: health care professional
MARS: Mobile App Rating Scale
mHealth: mobile health
TCM: traditional Chinese medicine
WHO: World Health Organization
