# Supplementary figures and images for: Comparing Smartphone Apps for Traditional Chinese Medicine and Modern Medicine in China: Systematic Search and Content Analysis
Source: JMIR Mhealth Uhealth. 2021 Mar 24;9(3):e27406. doi: 10.2196/27406 (PMC8108569; doi:10.2196/27406)

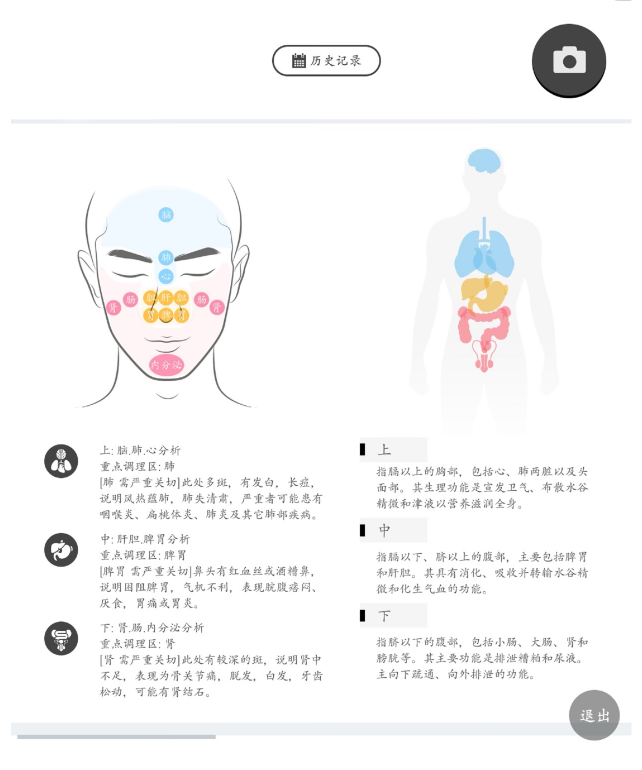

Supplement: Multimedia Appendix 1 [file mhealth_v9i3e27406_app1.png]
